# Effectiveness of modified hyper‐CVAD chemotherapy regimen in the treatment of adult acute lymphoblastic leukemia: a retrospective experience

**DOI:** 10.1002/cam4.1328

**Published:** 2018-01-31

**Authors:** Hasan Jalaeikhoo, Mohsen Rajaeinejad, Manoutchehr Keyhani, Mohammad Zokaasadi, Mohammad Mehdi Dehghani Firoozabadi

**Affiliations:** ^1^ AJA Cancer Epidemiology Research and Treatment Center (AJA‐ CERTC) AJA University of Medical Sciences Tehran Iran; ^2^ Hematology and Oncology Research Center Vali‐Asr Hospital Tehran University of Medical Sciences Tehran Iran

**Keywords:** Acute lymphoblastic leukemia, modified Hyper‐CVAD, survival analysis

## Abstract

Several chemotherapy regimens have been developed for the treatment of acute lymphoblastic leukemia (ALL), but relapse still presents the most common obstacles to attaining long‐term survival. The hyper‐CVAD (hyperfractionated cyclophosphamide, vincristine, doxorubicin, and prednisolone)/HD MTX and Ara‐C (high‐dose methotrexate and cytarabine) chemotherapy regimen was first started in the MD Anderson Cancer Center as an intensive regimen for adult patients with ALL. The purpose of this study was to evaluate the effectiveness of a modified hyper‐CVAD protocol. We used hyper‐CVAD as consolidation/maintenance after remission induction with daunorubicin, vincristine, and prednisolone (and cyclophosphamide for T‐cell ALL only) rather than standard hyper‐CVAD in order to reduce treatment complications. This study was conducted as a retrospective review of medical records of ALL patients at 501 army hospital, Tehran, Iran, from 2005 to 2015. Three hundred and one patients underwent modified hyper‐CVAD chemotherapy regimen. Complete remission and overall survival (OS) rates were measured as primary endpoints. Two hundred and forty‐six (81.7%) reached complete remission (CR) during the first 6 months of treatment, and 55 patients (18.3%) did not reach CR. The 5‐year OS rate was 51.8% (95% CI (confidence interval): 45.1–57.8%). Modified hyper‐CVAD regimen is an efficient intensive chemotherapy regimen for consolidation/maintenance of adults with newly diagnosed ALL and has an acceptable 5‐year overall that is comparable to standard hyper‐CVAD regimen.

## Introduction

Acute lymphoblastic leukemia (ALL) is a hematological malignancy with different pathological subtypes; Pre‐B‐cell ALL (the most common subtype), pre‐T‐cell ALL, T‐cell ALL, and B‐cell ALL 1. Several chemotherapy regimens have been used and developed in the treatment of ALL. The most effective chemotherapy regimen in the treatment of adult ALL is not yet clear.

The most widely used protocols were cancer and leukemia group B (CALGB) program which consisted of a five‐agent combination, Group for Research on Adult Acute Lymphoblastic Leukemia 2003 (GRAALL 2003) protocol and more recently hyper‐CVAD 2, 3. Moreover, the Berlin–Frankfurt–Munster (BFM) regimen was mainly used in pediatric ALL and can be considered for young adults with ALL 4.

The hyper‐CVAD regimen (hyperfractionated cyclophosphamide, vincristine, doxorubicin [Adriamycin], and dexamethasone)/HD MTX and Ara‐C (high‐dose methotrexate and cytarabine) are used widely in many institutions 5, 6. The MD Anderson Cancer Center (MDACC) study reported a complete response (CR) rate of 92%, 5‐year overall survival of 38% and progression‐free survival (PFS) of 66% for adult patients with ALL 7, 8.

The hyper‐CVAD regimen is made up of three curative steps: induction, consolidation, and subsequent maintenance therapy. In our study, in order to reduce treatment‐related toxicities, we developed a modified hyper‐CVAD regimen. This modified protocol has been used for more than a decade in our center as preliminary results were promising 9.

We modified the original hyper‐CVAD protocol in order to reduce treatment‐related complications of an intensive induction chemotherapy. After remission induction with daunorubicin, vincristine, and prednisolone (and cyclophosphamide only for T‐cell ALL), we proceeded to hyper‐CVAD regimen.

We analyzed the long‐term outcomes of modified hyper‐CVAD regimen in a relatively large sample in our cancer center.

## Materials and Methods

### Patient selection and ethical considerations

From 2005 to 2015, a total number of 301 patients have been diagnosed with adult ALL and treated using modified hyper‐CVAD protocol in our cancer center. Their medical records were selected for investigation. The study protocol was approved by ethical committee of AJA University of Medical Sciences.

### Diagnosis

The diagnosis of ALL was initially made by bone marrow examination (aspirate and biopsy). Involvement of 20% of bone marrow by malignant lymphoblasts or presence of them in peripheral blood was interpreted as a confirmed diagnosis. The presence of the disease in central nervous system (CNS) was documented when more than 5 lymphoblasts per mm^3^ were seen in the cerebrospinal fluid (CSF).

### Pretreatment

Before starting the induction phase, patients were evaluated for signs and symptoms of infection, renal failure, uremia, hyperuricemia, tumor lysis syndrome, and other concomitant medical conditions. Based on positive findings, they were treated, and the underlying abnormalities were rectified.

### Modification of standard hyper‐CVAD protocol

The modified hyper‐CVAD protocol included hyper‐CVAD as the consolidation/maintenance after remission induction with vincristine, daunorubicin, and prednisolone.

### Treatment protocol

Remission induction was the same for different ALL subtypes and consisted of following drugs: Daunorubicin 45 mg/m^2^ on days 1–3, Vincristine 2 mg on days 1, 8, 15, 22, and Prednisolone 60 mg/m^2^ on days 1–35. Another dose of daunorubicin was given to the patients at the 14th day of treatment if they showed signs of persistent leukemic involvement in the bone marrow evaluation. CR was defined as follows: absolute neutrophil count of >1000/*μ*L, platelet count of >100,000/*μ*L, independence from red blood cell transfusion, absence of extramedullary involvement, and a bone marrow blast of <5%. Details of consolidation/maintenance of treatment protocol are summarized in Table 1. Courses A and B as described in Table 1 were given to the patients for four times each, and G‐CSF was prescribed twice daily if WBC count was <2000/*μ*L and once daily if the count was <3000/*μ*L. The patients were treated with G‐CSF in the A course and B course interval (14‐ to 21‐day interval). The maintenance phase was as follows: Vincristine 1.4 mg/m^2^ (monthly), Prednisolone 100 mg/day (for 5 days), MTX 15–20 mg/m^2^ (once weekly), and 6‐mercaptopurine (6‐MP) 60 mg/m^2^ (once daily). BCR/ABL (+) patients received imatinib mesylate 400 mg daily for lifelong from the time it was available in Iran (2007). Maintenance was prolonged for 2 years. Indications for stem cell transplantation were Ph(+) and patients who were in second or more complete remission (CR2 or higher).

**Table 1:** Details of modified hyper‐CVAD protocol

Drug	Dosage and timing
Course A	
Cyclophosphamide	300 mg/m^2^/IV BD (1st to 3rd day)
Vincristine	2 mg/IV (4th and 11th days)
Doxorubicin	60 mg/m^2^/IV (4th day)
Dexamethasone	40 mg/day (1st to 4th day, 11th to 14th day)
Ranitidine	Tablet 150 mg/BD or Omeprazole 20 mg daily
Course B	
MTX	1000 mg/m^2^/IV (1st day)
Folinic acid	18 h after MTX (30 mg IV every 6 h for 3–5 days
Ara‐C	3000 mg/m^2^/IV (2nd and 3rd day)
Sodium bicarbonate	One vial before the initiation of MTX
CNS prophylaxis	
MTX	15–20 mg (2nd day)
Ara‐C	100 mg (2nd day)

### CNS prophylaxis and treatment

An intrathecal (IT) injection of Ara‐C 50 mg plus methotrexate (MTX) 15 mg was given even if there was no evidence of CNS involvement. The brain radiotherapy (RT) was not performed preemptively and was only preserved for cases of CNS involvement. Brain RT was administered for 10 courses or more (4500 rad). During the RT, cytosine arabinoside (Ara‐C) 50 mg/m^2^ was given via intrathecal injection to the patients. The IT injections were continued until the CSF became clear and then were sustained biweekly and then monthly.

### Statistical analysis

Primary endpoints of the current study were complete remission and overall survival. Overall survival was defined as the time elapsed from diagnosis to death from any cause. Kaplan–Meier method was used to estimate survival rates. Survival curves were compared by means of log rank test, and *P*‐values of <0.05 were considered statistically significant. All analyses were performed by SPSS software for Windows version 22 Armonk, NY.

## Results

Patients ranged from 14 to 73 years of age. Three hundred and one patients were treated by modified hyper‐CVAD. The median follow‐up time was 48 months. The demographics of patients are summarized in Table 2.

**Table 2:** Baseline characteristics

Covariate	Category	Description
Sex	Male	62.8% (*n* = 189)
	Female	37.2% (*n* = 112)
Age	<18 years	15.6% (*n* = 47)
	19–30	38.5% (*n* = 116)
	31–40	30.6% (*n* = 92)
	41–60	7% (*n* = 21)
	Over 60	8.3% (*n* = 25)
Median WBC		8800/*μ*L
BMT	Yes	4.3% (*n* = 13)
	No	95.7% (*n* = 288)
Brain radiotherapy	Yes	31.6% (*n* = 95)
	No	68.4% (*n* = 206)
Intrathecal	Yes	90.4% (*n* = 272)
	No	9.6% (*n* = 29)
Relapse	Yes	34.3% (81/236)*
	No	65.7% (155/236)^a^
Survival status	Alive	54.2% (*n* = 163)
	Dead	45.8% (*n* = 138)
Cause of death	Relapse or refractory disease	52.9% (*n* = 73)
	Chemotherapy toxic side effects	28.3% (*n* = 39)
	Infection	14.5% (*n* = 20)
	Other	4.3% (*n* = 6)
Relapse area	Brain	26.7% (*n* = 19)
	Bone marrow	59.2% (*n* = 42)
	Other	14.1% (*n* = 10)

a Percentages were calculated among those who survived enough to potentially have a disease relapse (At least until consolidation phase).

### Outcome of the modified hyper‐CVAD protocol

**Figure 1:**
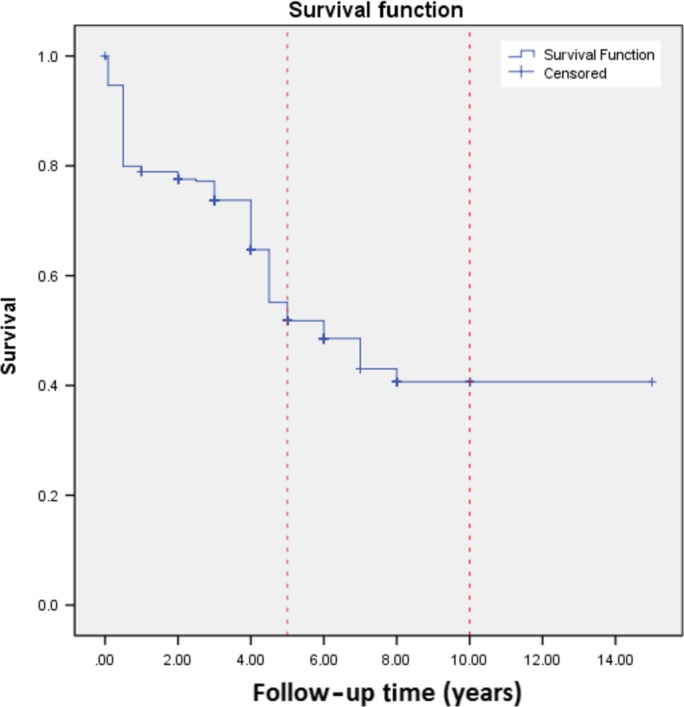
Kaplan–Meier curve for OS.

One hundred and eighty‐nine (62.8%) male and 112 (37.2%) female patients were treated by modified hyper‐CVAD. Two hundred and fifty‐five (84.7%) patients were younger than 40 years, and 46 (15.3%) patients were older than 40 years. Two hundred and forty‐six (81.7%) patients reached CR during the first 6 months of treatment. We observed that 55 (18.3%) patients did not reach the CR. We had 10 deaths and 81 relapses during the consolidation phase; of which 73 (90.1%) led to death. Up to the end of follow‐up time 163 patients were alive. The 5‐year overall survival (OS) of the patients was 51.8% (95% CI: 45.1‐57.8%), Figure 1.

**Figure 2:**
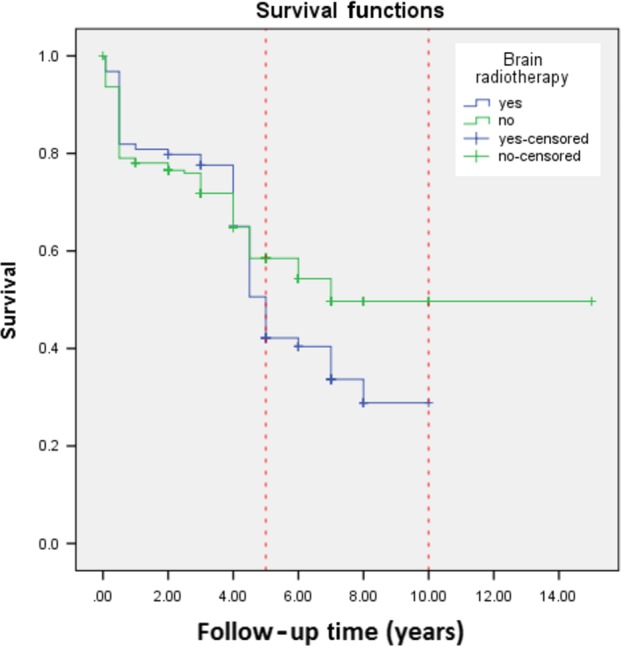
Survival curves based on receiving brain RT.

Ninety‐five (31.6%) patients received brain radiotherapy and 272 (90.4%) received intrathecal therapy. Survival analysis revealed that 5‐year OS was slightly higher in patients who did not receive RT, although the difference was not statistically significant (42.2% vs. 58.5%, *P* = 0.12), Figure 2.

**Figure 3:**
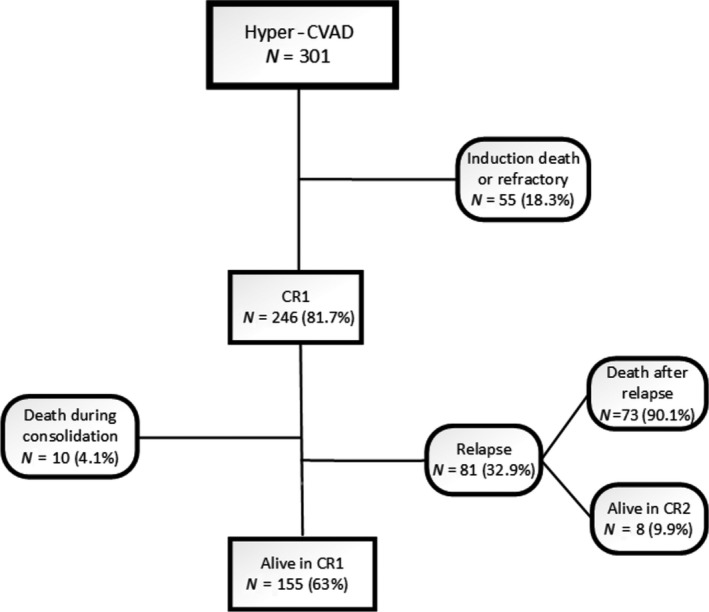
Schematic demonstration of modified hyper‐CVAD outcomes.

The presence of BCR/ABL gene has been evaluated in almost all of the patients, but only nine patients (~3%) were positive. When patients relapsed, they were offered BMT, but only 20 of 81 patients were able to undergo BMT. Some did not have suitable donors and some did not survive enough to reach the scheduled BMT. A flow diagram of the modified hyper‐CVAD regimen outcomes is depicted in Figure 3.

## Discussion

Despite advances in the treatment of acute lymphoblastic leukemia including different types of chimeric antigen receptor (CAR) T‐cells, monoclonal antibodies like epratuzumab and also bispecific T‐cell engagers (BiTEs) such as blinatumomab 10, its current state in Iran has not changed dramatically over the past 25 years, and outcomes remain disappointing because of low survival in older adults, very expensive care even after remission and relapses with no apparent reason. More intensive chemotherapy regimens have been administered for the ALL patients to overcome the disease, but they have greater toxicity and more side effects and therefore higher mortality rates. The hyper‐CVAD chemotherapy regimen was first introduced and developed in the MD Anderson Cancer Center (MDACC) 8. The MDACC protocol achieved a CR of 92% and 5‐year overall survival of 38%. We compared the results of modified hyper‐CVAD treatment in our cancer center with the results of previous studies. We observed that 81.6% of patients were in CR in the first 6 months and the 5‐year overall survival (OS) of the patients was 51.7% (95% CI: 45.1‐57.8%). This is somewhat better than similar protocols reported in the literature.

Although many chemotherapy regimens result in acceptable CR (about 90% in previous studies), the long‐term overall survival (OS) rate is still poor 5, 6, 11. The 5‐year disease‐free survival of the patients with different intensive chemotherapy regimens is <40% 1, 12, 13, 14. The relapse happens in the course of 1–3 months after CR. In addition, the immune deficiency of long duration induced by treatment causes very rare and/or catastrophic infections.These infections sometimes mimic relapse and place a tremendous psychological burden on families. Success of treatment depends mostly on effective eradication of tumor from bone marrow and other sites (mainly CNS). The course of hyper‐CVAD treatment is prolonged over 3–5 years and in the case of relapsed disease the maintenance will be even longer. Lengthy duration of maintenance chemotherapy makes it difficult for the patients to stay on the protocol and many patients cannot comply with it. This might have a negative impact on the treatment of acute leukemia in adults. More toxic regimens have been prescribed to adults with acute lymphoblastic leukemia to omit radiotherapy which has a strong negative impact on cognitive function and some other adverse effects as well 15. Shortening of the treatment duration with allogeneic bone marrow transplantation causes an excess mortality of about 20 percent 16, this seems unacceptable when added to the early mortality of remission induction, later mortality of consolidation, and late mortality of secondary cancers 17, 18 Our modified hyper‐CVAD protocol was less intensive and less toxic, thus easier to tolerate and part of the success of our strategy may have been patients' long‐term tolerance of this protocol.

The CR rate in our study (81.6%) was lower in comparison with the previous studies, but the higher 5‐year survival rate (51.7%) shows the efficacy and potential strength of this intensive chemotherapy regimen in the management of adult ALL patients. We administered the hyper‐CVAD as a consolidation/maintenance therapy; therefore, lower rates of CR due to less intensive induction and better survivals due to less toxicity were predictable to some extent. In a study published recently in Brazil, 49 patients underwent the hyper‐CVAD chemotherapy regimen, and the CR and 5‐year OS was 93.8 and 35%, respectively 6.

In another study that the hyper‐CVAD and Allogeneic hematopoietic stem cell transplantation (HSCT) were concomitantly carried out in ALL patients, the CR and 3‐year OS rate were 76.5 and 76%, respectively 5. In the Swedish study on patients with lymphoblastic lymphoma, the overall response rate was 97%, and the 5‐year progression‐free survival was 49% for the hyper‐CVAD protocol 11.

Our survival rate was higher than previously reported 5‐year survival rates. This is probably due to the early mortality of the standard hyper‐CVAD protocol. In our study, 81 relapses occurred after CR of which 73 of them led to death. Also we had 10 deaths during the consolidation phase in our modified hyper‐CVAD cohort and finally 163 patients alive during follow‐up time. The 5‐year overall survival of 51.8% is achieved in adult ALL patients in our center which can be comparable to stem cell transplantation 19. Better prospective and matched trials can evaluate the effect of possible treatment factors on the prognosis of adult ALL.

Finally, RT was performed for patients with CNS involvement in our modified hyper‐CVAD cohort, and the results showed a slightly better long‐term outcome in whom did not undergo brain RT. While this difference was not statistically different, it is likely that underlying differences in disease severity explained the finding. Patients who underwent brain radiotherapy might have more severe disease.

Our study has limitations that deserve to be mentioned. This is a retrospective report of survival, there is no control arm. It is possible that differences in the survival which we observed in comparison with previously reported studies are a result of a baseline differences in the populations studied. However, we had data on a larger sample of patients than most studies, another credible reason of higher survival rate for our patients is that because Ph (+) patients were just a limited number in our study (nine patients overall), thus the results of the long‐term survival are better compared to previous studies. We referred all Ph (+) patients for BMT but only four of them reached the treatment, and they all died after the BMT due to transplant complications.

Compared to other protocols, the modified hyper‐CVAD is an acceptable intensive chemotherapy regimen for the newly diagnosed adult PH (‐) ALL patients.

## Conflict of Interest

None declared.
